# The efficacy and safety of bevacizumab combined with FOLFOX regimen in the treatment of advanced colorectal cancer: A systematic review and meta-analysis

**DOI:** 10.1097/MD.0000000000026714

**Published:** 2021-07-30

**Authors:** Hailing Zhang, Jinzhi You, Wei Liu, Dandan Chen, Shiqi Zhang, Xiaoyan Wang

**Affiliations:** a Department of Gastroenterology, The Affiliated Suqian First People's Hospital of Nanjing Medical University, Suqian, China; b Department of Cardiothoracic Surgery, The Affiliated Suqian Hospital of Xuzhou Medical University, Suqian, China.

**Keywords:** bevacizumab, cancer, colorectal cancer, FOLFOX regimen, meta-analysis, treatment

## Abstract

**Background:**

It is necessary to systematically evaluate the clinical efficacy and safety of bevacizumab (BEV) combined with 5-fluorouracil + leucovorin + oxaliplatin (FOLFOX) regimen in the treatment of advanced colorectal cancer.

**Methods:**

We searched the PubMed et al databases for randomized controlled trials (RCTs) on the BEV combined with the FOLFOX regimen in the treatment of advanced colorectal cancer up to January 20, 2021. The Cochrane Collaborations’ risk of bias tool was used for the quality assessment of included RCTs. Revman5.3 software was used for meta-analysis.

**Results:**

Eleven RCTs with a total of 3178 patients with advanced colorectal cancer were included, meta-analysis results showed that the objective response rate (odds ratio [OR] = 3.15, 95% confidence intervals [CI]: 2.25–4.40, *P* < .001) and cancer control rate (OR = 2.73, 95% CI: 1.91–3.90, *P* < .001) of BEV + FOLFOX were higher than that of FOLFOX group. And the incidence of gastrointestinal adverse reactions (OR = 1.29, 95% CI: 1.07–1.55, *P* = .008) in the BEV + FOLFOX group was higher than that of the FOLFOX group, there were no significant differences in the incidence of leukopenia (OR = 1.04, 95% CI: 0.72–1.50, *P* = .83), hypertension (OR = 3.92, 95% CI: 0.81–18.88, *P* = .09) and neurotoxicity (OR = 1.00, 95% CI: 0.8–1.27, *P* = .98) between the 2 groups.

**Conclusion:**

BEV combined with the FOLFOX regimen is more effective than the FOLFOX regimen alone in the treatment of advanced colorectal cancer, but it may also increase the risk of gastrointestinal adverse reactions.

## 1 Introduction

Colorectal cancer is one of the most common malignant tumors of the digestive tract in the world.^[1]^ It has been reported that there were 97,220 new cases and 50,630 deaths related to colorectal cancer in the United States in 2018.^[2]^ In 2015, there were 376,300 new cases in China and 191,000 deaths related to colorectal cancer.^[3,4]^ The treatment of local colorectal cancer usually adopts surgery combined with adjuvant chemotherapy or combined with radiotherapy, but 50% to 55% of colorectal cancer patients metastasize during diagnosis or treatment.^[5]^ Chemotherapy for most patients can only improve the quality of life yet cannot prolong the survival period. When surgery is not feasible, the main treatment methods are chemotherapy and targeted therapy. Therefore, the effectiveness and safety of chemotherapy-related to colorectal cancer is on the top research agenda of colorectal cancer.^[6]^

The chemotherapy regimen of 5-fluorouracil + leucovorin + oxaliplatin (FOLFOX) is widely used in the treatment of advanced colorectal cancer.^[7]^ Bevacizumab (BEV) is a recombinant humanized monoclonal antibody of vascular endothelial growth factor (VEGF) A.^[8]^ It has been first approved by the US Food and Drug Administration for the treatment of metastatic colorectal cancer (mCRC) in 2004. It can inhibit the DNA replication of tumor endothelial cells and reduce tumor angiogenesis, thereby inhibiting tumor growth and exerting anti-tumor effects.^[9,10]^ At present, BEV combined with FOLFOX chemotherapy has been widely used in the treatment of advanced colorectal cancer, but there is a lack of relevant systematic reviews to evaluate the potential effects and safety. Therefore, we aimed to conduct a meta-analysis of randomized controlled trials (RCTs) on the BEV combined with FOLFOX regimen in the treatment of advanced colorectal cancer, to compare BEV combined with FOLFOX regimen and FOLFOX regimen alone in the treatment of advanced colorectal cancer, thereby providing reliable evidence for the clinical treatment of colorectal cancer.

## 2 Methods

We performed and reported this meta-analysis and systematic review in compliance with the Preferred Reporting Items for Systematic Reviews and Meta-Analyses.^[11]^

### 2.1 Literature search

Two authors independently searched the electronic databases including PubMed, EMBASE, Cochrane Library, China National Knowledge Infrastructure, Wanfang Database, and China Biomedical Literature Database. The research data were last updated on January 20, 2021. The following keywords and medical subject headings were used: “5-fluorouracil” or “leucovorin” or “oxaliplatin” or “FOLFOX” or “BEV” or “Bevacizumab” and “colorectal cancer” or “rectal” or “colon” or “oncology.” Reference lists of the relevant articles were also reviewed for any additional relevant studies.

### 2.2 Inclusion and exclusion criteria

The inclusion criteria of this meta-analysis were (1) the study population were patients with advanced colorectal cancer; (2) RCT study design comparing BEV combined with FOLFOX regimen and FOLFOX regimen alone; and (3) the language of the literature was reported in the Chinese or English. The exclusion criteria for this meta-analysis were (1) non-RCT studies; (2) repeated published studies, or studies with data not available for extraction; (3) different drug treatments; and (4) summary, comments of related topics were excluded.

### 2.3 Indicators

The clinical efficacy was divided into complete remission according to the evaluation criteria of chemotherapy efficacy for solid tumors established by the World Health Organization^[12]^: complete remission refers to the disappearance of all target lesions, no new lesions appeared, and the maintenance time exceeded 4 weeks; partial remission: the maximum diameter of the tumor is reduced by more than 30% compared with the basic value, and the maintenance time is more than 4 weeks; disease progression: the appearance of new lesions or the sum of the long diameters of the lesions increases by more than 20%, and the maintenance time is more than 4 weeks; stable status: the change of tumor lesions between partial remission and disease progression. Objective response rate = (complete response + partial response)/total number of cases × 100%, cancer control rate = (complete response + partial response + stable disease)/total number of cases × 100%. Furthermore, the related adverse outcome indicators were collected, including the incidence of gastrointestinal adverse reaction, incidence of leukopenia incidence of hypertension, and incidence of neurotoxicity.

### 2.4 Quality assessment

The Cochrane Collaborations’ risk of bias tool^[13]^ was used by 2 authors independently to evaluate the methodological quality and risk of bias of the included RCTs. Any disagreements were resolved by further discussion and consensus. The Cochrane Collaborations’ risk of bias tool included 7 specific domains, including sequence generation, allocation concealment, blinding of participants and personnel, blinding of outcome assessment, incomplete outcome data, selective outcome reporting, and other issues. Each domain could be rated as low risk of bias, high risk of bias, or unclear risk of bias based on the judging criteria.

### 2.5 Statistical methods

The statistical data was analyzed using Revman 5.3 software provided by the Cochrane Collaboration. The *Q* test was used to analyze the heterogeneity. If *P* > .1 and *I*^2^ < 50%, the fixed effects model was used. If *P* ≤ .1 and *I*^2^ ≥ 50%, the random-effects model was used. Binary outcomes were presented as Mantel–Haenszel-style odds ratios (ORs) with 95% confidence intervals (CI). Continuous outcomes were presented as mean differences. Additionally, the funnel chart was used to detect the publication bias of synthesized results. *P* < .05 indicated that the difference between groups was statistically significant.

## 3 Results

### 3.1 The study inclusion

**Figure 1:**
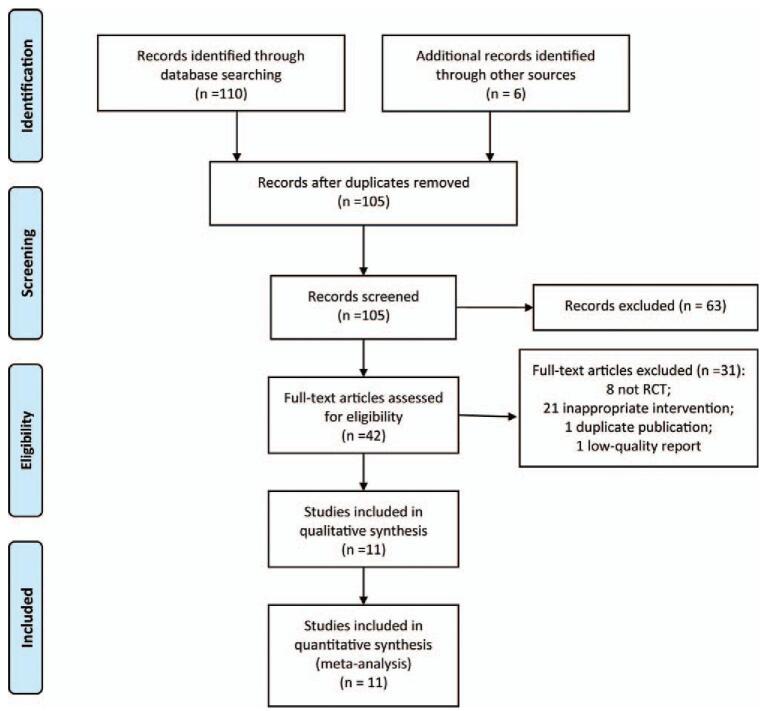
The PRISMA flow diagram of study selection. PRISMA = Preferred Reporting Items for Systematic Reviews and Meta-Analyses.

The initial search yielded 116 potentially relevant articles. Of these identified articles, 11 studies were excluded as duplicates. After viewing the titles and abstracts of the 105 remaining studies, the full texts of 42 studies were retrieved. Among them, 31 RCTs were excluded with failure to meet the inclusion criteria. Finally, 11 RCTs^[14–24]^ were included for data synthesized analysis. The process of study selection was presented in Figure 1.

### 3.2 Features of included RCTs

The 11 RCTs included a total of 3178 patients with colorectal cancer, including 1599 patients in the BEV + FOLFOX group and 1579 patients in the FOLFOX group. The included 11 studies included a total of 3178 patients with colorectal cancer, including 1599 patients in the BEV + FOLFOX group and 1579 patients in the FOLFOX group. The characteristics of included RCTs were presented in Table 1.

**Table 1:** The characteristics of included RCTs.

		Sample size		Treatment		Complete remission (cases)		Partial remission (cases)		Disease progression (cases)		Stable status (cases)	
Study	Country	BEV group (male/female)	BEV + FOLFOX group (male/female)	BEV group	BEV + FOLFOX group	BEV group	BEV + FOLFOX group	BEV group	BEV + FOLFOX group	BEV group	BEV + FOLFOX group	BEV group	BEV + FOLFOX group
Saifuddin 2015	China	25	25	Bevacizumab 5 mg/kg + FOLFOX6	FOLFOX6 (on the first day, oxaliplatin 100 mg/m^2^ was given intravenously for 3 h; then 5-fluorouracil was given 400 mg/m^2^, on the first day, and then 2400 mg/m^2^, continuously intravenously for 46 h, 5-fluorouracil was given calcium leucovorin, 400 mg/m^2^, intravenously for 2 h, on the first day. The above therapies were repeated every 2 weeks.)	0	0	14	10	3	6	8	9
Lu 2014	China	18/12	17/13	Bevacizumab 5 mg/kg + FOLFOX	FOLFOX (oxaliplatin 85 mg/m^2^, intravenous infusion for 2 h, d1; leucovorin (400 mg/m^2^, intravenous infusion for 2 h, d1; 5-fluorouracil 400 mg/m^2^, intravenous infusion, d1; 5-fluorouracil 2 400 mg/m^2^, infusion 46 h, d1. Every 14 days was a treatment cycle.)	3	1	10	4	3	8	14	17
Xiao 2016	China	27/18	28/17	Bevacizumab 7.5 mg/kg + FOLFOX	FOLFOX (oxaliplatin 85 mg m^−2^, intravenous infusion for 2 h, d1; 5-fluorouracil 400 mg m^−2^, intravenous infusion for 2 h, d1, and then 2400 mg m^−2^ continuous intravenous infusion 46 h; leucovorin calcium 400 mg m^−2^, intravenous infusion for 2 h, d1. 14 days was a chemotherapy cycle.)	1	0	33	22	10	19	1	4
Lu 2017	China	43/31	43/31	Bevacizumab 5 mg/kg + FOLFOX	FOLFOX (oxaliplatin 85 mg/m^2^, intravenous infusion for 2 h, d1; leucovorin (400 mg/m^2^, intravenous infusion for 2 h, d1; 5-fluorouracil 400 mg/m^2^, intravenous infusion, d1; 5-fluorouracil 2 400 mg/m^2^, infusion 46 h, d1. Every 14 days was a treatment cycle.)	8	2	23	9	14	31	29	32
Yong 2017	China	16/13	17/12	Bevacizumab 7.5 mg/kg + FOLFOX6	FOLFOX6 (oxaliplatin 85 mg m^−2^ + calcium leucovorin 200 mg m^−2^ + fluorouracil 400 mg m^−2^, once every 2 weeks, twice as a course of treatment; the test group is based on the control group, plus With bevacizumab 10 mg kg^−1^, start intravenous drip on the second day after the end of chemotherapy, at least 4 times, once every 2 weeks.)	2	1	13	6	2	7	12	15
Shi 2017	China	20/15	19/16	Bevacizumab 5 mg/kg + FOLFOX6	FOLFOX6 (on the first day, oxaliplatin 100 mg/m^2^ was given intravenously for 3 h; then 5-fluorouracil was given 400 mg/m^2^, on the first day, and then 2400 mg/m^2^, continuously intravenously for 46 h, 5-fluorouracil was given calcium leucovorin, 400 mg/m^2^, intravenously for 2 h, on the first day. The above therapies were repeated every 2 weeks.)	0	0	14	6	6	15	15	14
Si 2017	China	22/15	30/25	Bevacizumab 7.5 mg/kg + FOLFOX6	FOLFOX6 (oxaliplatin 85 mg m^−2^ + calcium leucovorin 200 mg m^−2^ + fluorouracil 400 mg m^−2^, once every 2 weeks, twice as a course of treatment; the test group is based on the control group, plus with bevacizumab 10 mg kg^−1^, start intravenous drip on the second day after the end of chemotherapy, at least 4 times, once every 2 weeks.)	12	4	30	17	3	8	10	8
Liao 2018	China	19/15	18/16	Bevacizumab 7.5 mg/kg + FOLFOX	FOLFOX (oxaliplatin 85 mg m^−2^, intravenous infusion for 2 h, d1; 5-fluorouracil 400 mg m^−2^, intravenous infusion for 2 h, d1, and then 2400 mg m^−2^ continuous intravenous infusion 46 h; leucovorin calcium 400 mg m^−2^, intravenous infusion for 2 h, d1. 14 days is a chemotherapy cycle.)	0	0	15	7	6	15	13	12
Xie 2015	China	18/15	17/14	Bevacizumab 5 mg/kg + FOLFOX	FOLFOX (oxaliplatin 85 mg/m^2^, intravenous infusion for 2 h, d1; leucovorin (400 mg/m^2^, intravenous infusion for 2 h, d1; 5-fluorouracil 400 mg/m^2^, intravenous infusion, d1; 5-fluorouracil 2 400 mg/m^2^, infusion 46 h, d1. Every 14 days is a treatment cycle.)	2	0	11	6	9	12	9	15
Luo 2018	China	53/33	55/31	Bevacizumab 5 mg/kg + FOLFOX	FOLFOX	-	-	-	-	-	-	-	-
Gramont 2012	France	656/495	587/568	Bevacizumab 5 mg/kg + FOLFOX4	FOLFOX4 (oxaliplatin 85 mg/m^2^, leucovorin 200 mg/m^2^, and fluorouracil 400 mg/m^2^ bolus plus 600 mg/m^2^ 22-h continuous infusion on day 1; leucovorin 200 mg/m^2^ plus fluorouracil 400 mg/m^2^ bolus plus 600 mg/m^2^ 22-h continuous infusion on day 2) every 2 weeks for 12 cycles)	-	-	-	-	-	-	-	-

### 3.3 Quality of included RCTs

**Figure 2:**
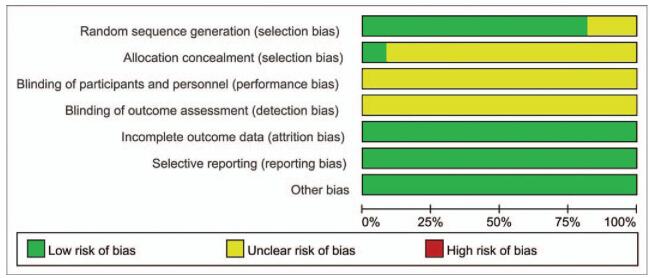
Risk of bias graph.

**Figure 3:**
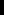
Risk of bias summary.

As indicated in Figures 2 and 3, although all of the included RCTs mentioned randomization, 2 RCTs^[21,23]^ did not provide a detailed description of the methods used for generating random sequences. Furthermore, only 1 study^[14]^ reported allocation blinding, all resting included RCTs did not report allocation blinding or the personnel blinding. For the blinding of outcome assessment, all included studies did not report the related information. No selective reporting or other significant biases amongst the 11 included RCTs were found.

### 3.4 Synthesized analysis

#### 3.4.1 Objective response rate

**Figure 4:**
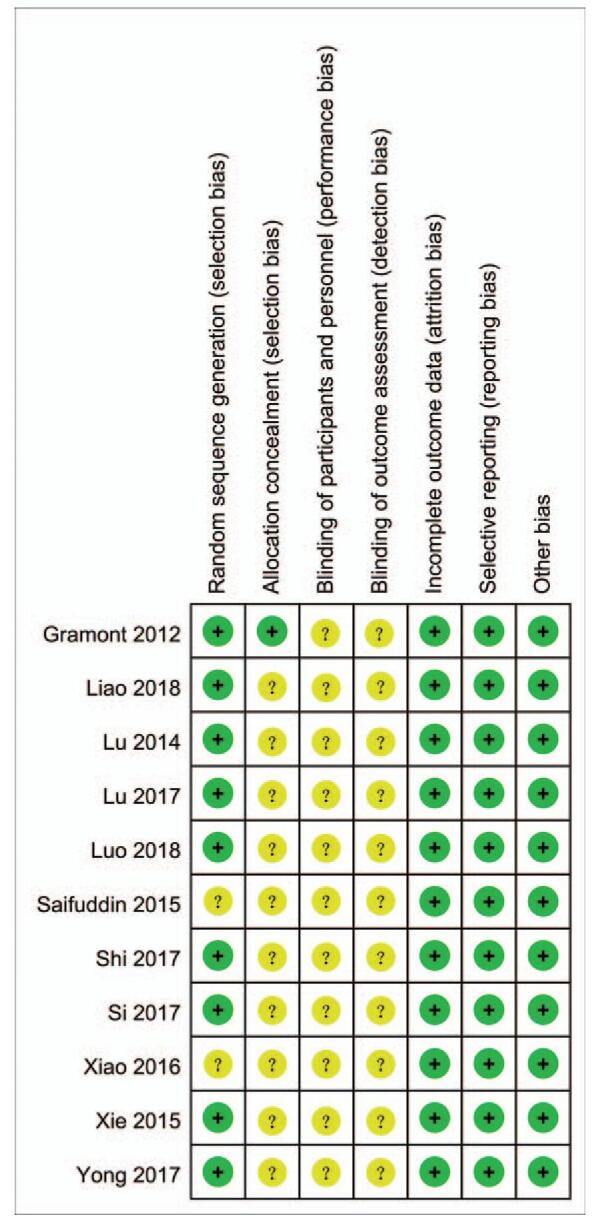
The forest plots for the synthesized objective response rate and cancer control rate.

A total of 9 studies reported the objective response rate during treatment, and the total number of cases was 700. Among them, there were 358 patients in the BEV + FOLFOX group, a total of 191 patients achieved objective remission, and 342 cases in the FOLFOX group, a total of 95 patients achieved objective remission. There was no heterogeneity among the included studies, so the fixed effects model was used. Meta-analysis results showed that the objective response rate of the BEV + FOLFOX group was higher than that of the FOLFOX group alone, and the difference was statistically significant (OR = 3.15, 95% CI: 2.25–4.40, *P* < .00, Fig. 4A).

#### 3.4.2 Cancer control rate

A total of 9 studies reported the cancer control rate during treatment, and the total number of cases was 900. Among them, there were 358 patients in the BEV + FOLFOX group, a total of 297 patients achieved cancer control rate, and 342 cases in the FOLFOX group, a total of 218 patients achieved cancer control rate. There was no heterogeneity among the included studies, so the fixed effects model was used. Meta-analysis results showed that the cancer control rate of the BEV + FOLFOX group was higher than that of the FOLFOX group alone, and the difference was statistically significant (OR = 2.73, 95% CI:1.91–3.90, *P* < .001, Fig. 4B).

#### 3.4.3 Incidence of gastrointestinal adverse reaction

**Figure 5:**
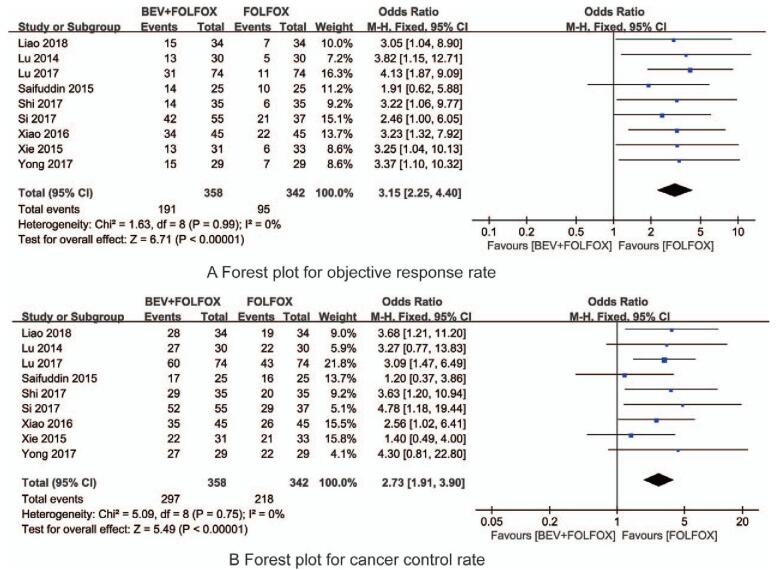
The forest plots for the safety-related outcomes.

Gastrointestinal reaction is one of the most common adverse reactions during chemotherapy, mainly manifested as nausea, vomiting, abdominal distension, diarrhea, anorexia, and indigestion. In this meta-analysis, 11 studies all reported the occurrence of gastrointestinal adverse reactions during treatment. The sample size was 3178 patients, of which 1599 cases occurred in the BEV + FOLFOX group, and 389 cases had gastrointestinal adverse reactions, while the FOLFOX group had 1579 cases, and of which 316 cases had gastrointestinal adverse reactions. The homogeneity between the included 11 RCTS was small, so the fixed effects model was adopted. Meta-analysis results showed that BEV could increase the incidence of gastrointestinal reactions in patients with advanced colorectal cancer (OR = 1.29, 95% CI: 1.07–1.55, *P* = .008, Fig. 5A).

#### 3.4.4 Incidence of leukopenia

Chemotherapy drugs would have a certain degree of bone marrow suppression, so it might be accompanied by a certain degree of leukopenia. A total of 8 RCTs reported the incidence of leukopenia during chemotherapy with BEV and/or FOLFOX regimens. The 8 RCTs included 740 patients with advanced colorectal cancer, of which there were 379 patients in the BEV + FOLFOX group, 111 patients had leukopenia, 361 patients in the FOLFOX group, and 106 patients had leukopenia. The heterogeneity was small, therefore, a fixed-effect model was used. The results indicated that there was no significant difference in the incidence of leukopenia between the BEV + FOLFOX group and the FOLFOX group (OR = 1.04, 95% CI: 0.72–1.50, *P* = .83, Fig. 5B).

#### 3.4.5 Incidence of hypertension

A total of 6 RCTs reported the incidence of hypertension during chemotherapy. The 6 articles contained 2704 patients with advanced colorectal cancer, including 1368 in the BEV + FOLFOX group, of which 164 with hypertension, and 1346 in the FOLFOX group, 43 cases had hypertension. There was significant heterogeneity amongst the 6 included studies, so the random effects model was adopted. The results indicated that there was no significant difference in the incidence of hypertension between the BEV + FOLFOX group and the FOLFOX group (OR = 3.92, 95% CI: 0.81–18.88, *P* = .09, Fig. 5C).

#### 3.4.6 Incidence of neurotoxicity

A total of 6 studies = reported the number of patients with hypertension during chemotherapy. A total of 4 RCTs reported neurotoxicity during chemotherapy. The 4 RCTs contained 2492 patients with advanced colon cancer. There was no homogeneity amongst the included RCTs, then a fixed-effect model was used. Meta-analysis results showed that there was no statistically significant difference between the BEV + FOLFOX group and the FOLFOX group (OR = 1.00, 95% CI: 0.8–1.27, *P* = .98, Fig. 5D).

### 3.5 Publication bias analysis

**Figure 6:**
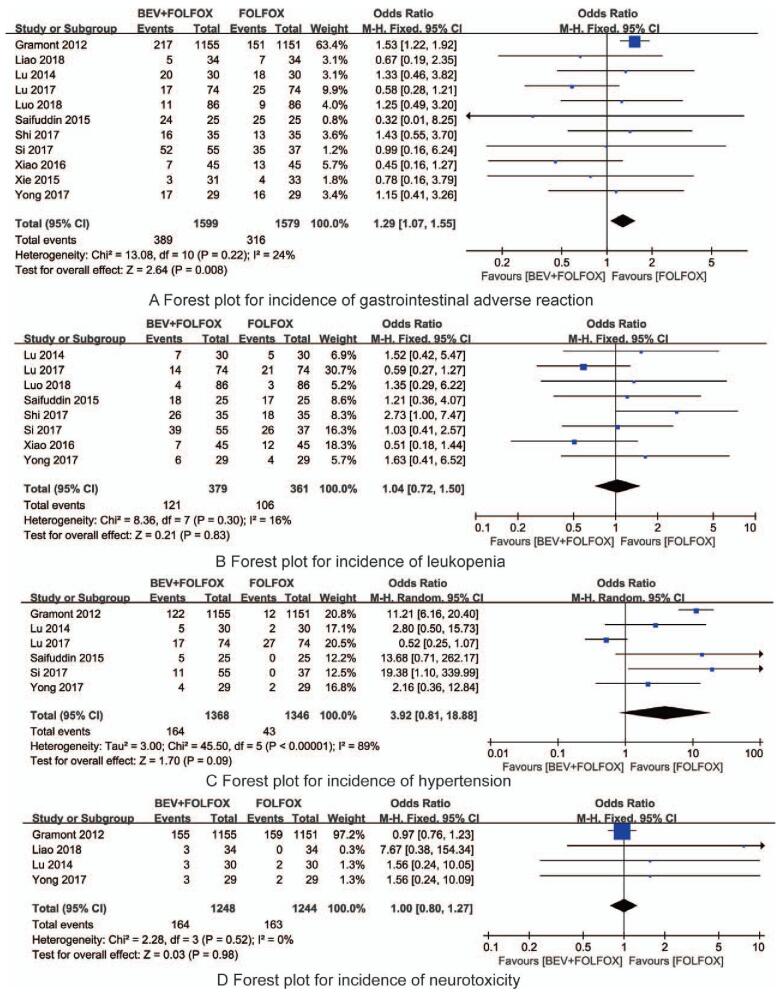
The funnel plots for the synthesized outcomes.

We evaluated publication bias using a funnel plot. As presented in Figure 6, the dots were evenly distributed in the funnel plots, and Egger tests indicated that there was no publication bias (all *P* > .05).

Sensitivity analyses, which investigate the influence of 1 study on the overall risk estimate by removing 1 study in each turn, suggested that the overall risk estimates were not substantially changed by any single study.

## 4 Discussion

It is been reported that the mortality of colorectal cancer ranks second in malignant tumors, second only to lung cancer.^[25]^ In China, the incidence of colorectal cancer is also increasing year by year.^[26]^ When cancer cannot be removed radically or distant metastasis occurs, the first choice is chemotherapy.^[27]^ However, chemotherapy has its drawbacks. Only 30% of patients can achieve the expected therapeutic effect.^[28]^ Chemotherapy has certain effects on tumor cells and normal cells, so it will lead to a series of adverse reactions, such as neutropenia, anemia, and hematopoietic dysfunction. With the discovery of many cellular molecular targets, a large number of selectively targeted drugs have been produced, which has opened up a new era for cancer treatment. These drugs target the inherent abnormalities of cancer cells and may be less toxic than traditional non-selective cytotoxic drugs. BEV is the first monoclonal antibody used for the treatment of advanced colorectal cancer, which can specifically bind to VEGF, thereby inhibiting the production of vascular endothelial growth.^[29,30]^ BEV brings new hope to the clinical treatment of cancer patients due to its advantages such as strong targeting, good curative effect, and few adverse reactions.^[31]^ The results of our meta-analysis results have shown that the objective response rate and cancer control rate of the combined group were higher than those of the control group, indicating that the BEV + FOLFOX regimen is more effective than the single FOLFOX regimen in the treatment of advanced colorectal cancer. In terms of adverse reactions, the incidence of gastrointestinal reactions in the BEV + FOLFOX group was higher than that of the FOLFOX group, other adverse reactions, such as leukopenia, hypertension, and neurotoxicity, were not significantly different between the 2 groups.

VEGF plays an important role in the process of angiogenesis. It can be expressed in normal cells, but it is expressed at high levels in tumors of various systems.^[32]^ As a VEGF receptor blocker, anti-VEGF can inhibit the growth of vascular endothelial cells and deprive the tumor of blood supply to inhibit tumor growth.^[33,34]^ BEV is a full-length recombinant monoclonal antibody that can bind to all subtypes of VEGF.^[35]^ It was approved by the US Food and Drug Administration in 2004 and has been successfully used in anti-tumor therapy.^[36]^ Previous studies^[37–39]^ have found that Rinotecan combined with BEV can significantly improve overall survival (OS) and objective response rate in the treatment of different cancers.

The FOLFOX + BEV regimen is stopped often due to prominent oxaliplatin-related adverse reactions. Vaidyanathan et al^[40]^ have adopted a “stop and go” program to reduce oxaliplatin-related adverse reactions, that is, use the BEV + FOLFOX program for 8 cycles to stop oxaliplatin when intolerance occurs, and continue to use 5-FU, leucovorin calcium combined with BEV regimen until the disease progresses, and then the BEV + FOLFOX regimen is applied on the basis of the disease progression until the second level of neurotoxicity is discontinued. Among 67 patients with mCRC, the overall response rate was 58%, the median progression-free survival (PFS) was 10.6 months, and the median OS was 26.7 months; the median progression time of the BEV + FOLFOX regimen group was 9.9 months, and the median OS was 23.2 months, oxaliplatin-related toxicity and treatment-related costs were reduced. Okita et al^[41]^ applied this program to 50 patients with mCRC, with an overall response rate of 48%, including 1 complete response and 23 partial responses. The 50 patients had a median follow-up time of 27.8 months with a median PFS of 12.8 months and a median OS of 30.1 months, comet to the finding that oxaliplatin-related toxic reactions were effectively reduced, confirming the safety and effectiveness of this regimen. However, this program still needs more clinical studies to prove its feasibility.

This study also has certain shortcomings that should be concerned. Firstly, the quality of the included articles is not high, and there is a lack of detailed descriptions of allocation concealment and blinding, future studies with rigorous design are needed. Secondly, the included studies lack the data of indicators such as OS and PFS, which we could not include for synthesized analysis. Thirdly, since included studies did not detect the genotypes of patients with RAS and BRAF, which are closely related to targeted therapy, it is impossible to further analyze the relationship between genotype and chemotherapy, future studies on the potential relationship between genotype and chemotherapy are warranted.

## 5 Conclusions

In conclusion, compared to the FOLFOX regimen alone, the BEV + FOLFOX regimen has a better effect in the treatment of colorectal cancer, but it will also increase the risk of gastrointestinal reactions to a certain extent. In addition, there was no statistically significant difference in the incidence of other adverse reactions between the 2 groups. Therefore, for patients with advanced colorectal cancer, BEV combined with the FOLOFX regimen can be selected for chemotherapy, and corresponding symptomatic supportive treatments for gastrointestinal reactions that occur can be given, which can improve the efficacy and reduce the risk of adverse reactions. However, due to the limited quality of the RCTs, the small sample size, and the lack of survival benefit indicators such as OS and PFS, a large sample and high-quality focused on the clinical efficacy and safety of the BEV + FOLFOX regimen in the treatment of advanced colorectal cancer are needed in the future.

## Author contributions

XW, HZ, and JY designed research; HZ, JY, WL, DC, and SZ conducted research; HZ and JY analyzed data; JY wrote the first draft of the manuscript; XW had primary responsibility for final content. All authors read and approved the final manuscript.

**Conceptualization:** Hailing Zhang, Wei Liu.

**Data curation:** Hailing Zhang, Dandan Chen, Xiaoyan Wang.

**Formal analysis:** Hailing Zhang, Jinzhi You, Wei Liu, Dandan Chen, Xiaoyan Wang.

**Funding acquisition:** Xiaoyan Wang.

**Investigation:** Hailing Zhang, Jinzhi You, Dandan Chen, Shiqi Zhang, Xiaoyan Wang.

**Methodology:** Hailing Zhang, Jinzhi You, Dandan Chen, Shiqi Zhang.

**Project administration:** Jinzhi You, Dandan Chen, Shiqi Zhang, Xiaoyan Wang.

**Resources:** Hailing Zhang, Wei Liu.

**Software:** Wei Liu, Shiqi Zhang.

**Supervision:** Shiqi Zhang, Xiaoyan Wang.

**Validation:** Hailing Zhang.

**Visualization:** Dandan Chen, Shiqi Zhang.

**Writing – original draft:** Hailing Zhang, Dandan Chen, Xiaoyan Wang.
